# Supplementary material for: Resolving the full spectrum of human genome variation using Linked-Reads
Source: Genome Res. 2019 Apr;29(4):635–45. doi: 10.1101/gr.234443.118 (PMC6442396; doi:10.1101/gr.234443.118)
Supplement: Supplemental Material [file supp_gr.234443.118_Supplemental_longranger-2.2.zip › longranger-2.2.tar.bz2!/lr2.2-public/lib/stan/cmdstan/stan/doc/api/html/agrad_2rev_2matrix_2quad__form_8hpp_source.html]

Stan: src/stan/agrad/rev/matrix/quad\_form.hpp Source File


|  |  |
| --- | --- |
|  | Stan  2.5.0  probability, sampling & optimization |


- Main Page
- Related Pages
- Namespaces
- Classes
- Files

- File List
- File Members

All Classes Namespaces Files Functions Variables Typedefs Enumerations Enumerator Friends Macros Pages

- src
- stan
- agrad
- rev
- matrix

quad\_form.hpp

Go to the documentation of this file.

1 #ifndef STAN\_\_AGRAD\_\_REV\_\_MATRIX\_\_QUAD\_FORM\_HPP

2 #define STAN\_\_AGRAD\_\_REV\_\_MATRIX\_\_QUAD\_FORM\_HPP

3

4 #include <boost/utility/enable\_if.hpp>

5 #include <boost/type\_traits.hpp>

6 #include <stan/math/matrix/Eigen.hpp>

7 #include <stan/math/matrix/typedefs.hpp>

8 #include <stan/agrad/rev.hpp>

9 #include <stan/agrad/rev/matrix/typedefs.hpp>

10 #include <stan/agrad/rev/matrix/value\_of.hpp>

11 #include <stan/math/matrix/quad\_form.hpp>

12 #include <stan/math/error\_handling/matrix/check\_multiplicable.hpp>

13 #include <stan/math/error\_handling/matrix/check\_square.hpp>

14 #include <stan/math/error\_handling/matrix/check\_symmetric.hpp>

15

16 namespace stan {

17  namespace agrad {

18  namespace {

19  template<typename TA,int RA,int CA,typename TB,int RB,int CB>

20  class quad\_form\_vari\_alloc : public chainable\_alloc {

21  private:

22  inline void compute(const Eigen::Matrix<double,RA,CA> &A,

23  const Eigen::Matrix<double,RB,CB> &B)

24  {

25  int i,j;

26  Eigen::Matrix<double,CB,CB> Cd(B.transpose()\*A\*B);

27  for (j = 0; j < C\_.cols(); j++) {

28  for (i = 0; i < C\_.rows(); i++) {

29  if (\_sym) {

30  C\_(i,j) = var(new vari(0.5\*(Cd(i,j) + Cd(j,i)),false));

31  }

32  else {

33  C\_(i,j) = var(new vari(Cd(i,j),false));

34  }

35  }

36  }

37  }

38

39  public:

40  quad\_form\_vari\_alloc(const Eigen::Matrix<TA,RA,CA> &A,

41  const Eigen::Matrix<TB,RB,CB> &B,

42  bool symmetric = false)

43  : A\_(A), B\_(B), C\_(B\_.cols(),B\_.cols()), \_sym(symmetric)

44  {

45  compute(value\_of(A),value\_of(B));

46  }

47

48  Eigen::Matrix<TA,RA,CA> A\_;

49  Eigen::Matrix<TB,RB,CB> B\_;

50  Eigen::Matrix<var,CB,CB> C\_;

51  bool \_sym;

52  };

53

54  template<typename TA,int RA,int CA,typename TB,int RB,int CB>

55  class quad\_form\_vari : public vari {

56  protected:

57  inline void chainA(Eigen::Matrix<double,RA,CA> &A,

58  const Eigen::Matrix<double,RB,CB> &Bd,

59  const Eigen::Matrix<double,CB,CB> &adjC) {}

60  inline void chainB(Eigen::Matrix<double,RB,CB> &B,

61  const Eigen::Matrix<double,RA,CA> &Ad,

62  const Eigen::Matrix<double,RB,CB> &Bd,

63  const Eigen::Matrix<double,CB,CB> &adjC) {}

64

65  inline void chainA(Eigen::Matrix<var,RA,CA> &A,

66  const Eigen::Matrix<double,RB,CB> &Bd,

67  const Eigen::Matrix<double,CB,CB> &adjC)

68  {

69  int i,j;

70  Eigen::Matrix<double,RA,CA> adjA(Bd\*adjC\*Bd.transpose());

71  for (j = 0; j < A.cols(); j++) {

72  for (i = 0; i < A.rows(); i++) {

73  A(i,j).vi\_->adj\_ += adjA(i,j);

74  }

75  }

76  }

77  inline void chainB(Eigen::Matrix<var,RB,CB> &B,

78  const Eigen::Matrix<double,RA,CA> &Ad,

79  const Eigen::Matrix<double,RB,CB> &Bd,

80  const Eigen::Matrix<double,CB,CB> &adjC)

81  {

82  int i,j;

83  Eigen::Matrix<double,RA,CA> adjB(Ad\*Bd\*adjC.transpose() + Ad.transpose()\*Bd\*adjC);

84  for (j = 0; j < B.cols(); j++)

85  for (i = 0; i < B.rows(); i++)

86  B(i,j).vi\_->adj\_ += adjB(i,j);

87  }

88

89  inline void chainAB(Eigen::Matrix<TA,RA,CA> &A,

90  Eigen::Matrix<TB,RB,CB> &B,

91  const Eigen::Matrix<double,RA,CA> &Ad,

92  const Eigen::Matrix<double,RB,CB> &Bd,

93  const Eigen::Matrix<double,CB,CB> &adjC)

94  {

95  chainA(A,Bd,adjC);

96  chainB(B,Ad,Bd,adjC);

97  }

98

99  public:

100  quad\_form\_vari(const Eigen::Matrix<TA,RA,CA> &A,

101  const Eigen::Matrix<TB,RB,CB> &B,

102  bool symmetric = false)

103  : vari(0.0) {

104  \_impl = new quad\_form\_vari\_alloc<TA,RA,CA,TB,RB,CB>(A,B,symmetric);

105  }

106

107  virtual void chain() {

108  int i,j;

109  Eigen::Matrix<double,CB,CB> adjC(\_impl->C\_.rows(),\_impl->C\_.cols());

110

111  for (j = 0; j < \_impl->C\_.cols(); j++)

112  for (i = 0; i < \_impl->C\_.rows(); i++)

113  adjC(i,j) = \_impl->C\_(i,j).vi\_->adj\_;

114

115  chainAB(\_impl->A\_, \_impl->B\_,

116  value\_of(\_impl->A\_), value\_of(\_impl->B\_),

117  adjC);

118  };

119

120  quad\_form\_vari\_alloc<TA,RA,CA,TB,RB,CB> \*\_impl;

121  };

122  }

123

124  template<typename TA,int RA,int CA,typename TB,int RB,int CB>

125  inline typename

126  boost::enable\_if\_c< boost::is\_same<TA,var>::value ||

127  boost::is\_same<TB,var>::value,

128  Eigen::Matrix<var,CB,CB> >::type

129  quad\_form(const Eigen::Matrix<TA,RA,CA> &A,

130  const Eigen::Matrix<TB,RB,CB> &B)

131  {

132  stan::math::check\_square("quad\_form(%1%)",A,"A",(double\*)0);

133  stan::math::check\_multiplicable("quad\_form(%1%)",A,"A",

134  B,"B",(double\*)0);

135

136  quad\_form\_vari<TA,RA,CA,TB,RB,CB> \*baseVari = new quad\_form\_vari<TA,RA,CA,TB,RB,CB>(A,B);

137

138  return baseVari->\_impl->C\_;

139  }

140  template<typename TA,int RA,int CA,typename TB,int RB>

141  inline typename

142  boost::enable\_if\_c< boost::is\_same<TA,var>::value ||

143  boost::is\_same<TB,var>::value,

144  var >::type

145  quad\_form(const Eigen::Matrix<TA,RA,CA> &A,

146  const Eigen::Matrix<TB,RB,1> &B)

147  {

148  stan::math::check\_square("quad\_form(%1%)",A,"A",(double\*)0);

149  stan::math::check\_multiplicable("quad\_form(%1%)",A,"A",

150  B,"B",(double\*)0);

151

152  quad\_form\_vari<TA,RA,CA,TB,RB,1> \*baseVari = new quad\_form\_vari<TA,RA,CA,TB,RB,1>(A,B);

153

154  return baseVari->\_impl->C\_(0,0);

155  }

156

157  template<typename TA,int RA,int CA,typename TB,int RB,int CB>

158  inline typename

159  boost::enable\_if\_c< boost::is\_same<TA,var>::value ||

160  boost::is\_same<TB,var>::value,

161  Eigen::Matrix<var,CB,CB> >::type

162  quad\_form\_sym(const Eigen::Matrix<TA,RA,CA> &A,

163  const Eigen::Matrix<TB,RB,CB> &B)

164  {

165  stan::math::check\_square("quad\_form(%1%)",A,"A",(double\*)0);

166  stan::math::check\_symmetric("quad\_form\_sym(%1%)",A,"A",(double\*)0);

167  stan::math::check\_multiplicable("quad\_form\_sym(%1%)",A,"A",

168  B,"B",(double\*)0);

169

170  quad\_form\_vari<TA,RA,CA,TB,RB,CB> \*baseVari = new quad\_form\_vari<TA,RA,CA,TB,RB,CB>(A,B,true);

171

172  return baseVari->\_impl->C\_;

173  }

174  template<typename TA,int RA,int CA,typename TB,int RB>

175  inline typename

176  boost::enable\_if\_c< boost::is\_same<TA,var>::value ||

177  boost::is\_same<TB,var>::value,

178  var >::type

179  quad\_form\_sym(const Eigen::Matrix<TA,RA,CA> &A,

180  const Eigen::Matrix<TB,RB,1> &B)

181  {

182  stan::math::check\_square("quad\_form(%1%)",A,"A",(double\*)0);

183  stan::math::check\_symmetric("quad\_form\_sym(%1%)",A,"A",(double\*)0);

184  stan::math::check\_multiplicable("quad\_form\_sym(%1%)",A,"A",

185  B,"B",(double\*)0);

186

187  quad\_form\_vari<TA,RA,CA,TB,RB,1> \*baseVari = new quad\_form\_vari<TA,RA,CA,TB,RB,1>(A,B,true);

188

189  return baseVari->\_impl->C\_(0,0);

190  }

191  }

192 }

193

194 #endif

\_impl

quad\_form\_vari\_alloc< TA, RA, CA, TB, RB, CB > \* \_impl

**Definition:** quad\_form.hpp:118

check\_multiplicable.hpp

typedefs.hpp

\_sym

bool \_sym

**Definition:** quad\_form.hpp:51

typedefs.hpp

C\_

Eigen::Matrix< var, CB, CB > C\_

**Definition:** quad\_form.hpp:50

quad\_form.hpp

stan::agrad::value\_of

T value\_of(const fvar< T > &v)

Return the value of the specified variable.

**Definition:** value\_of.hpp:16

check\_symmetric.hpp

stan::math::check\_symmetric

bool check\_symmetric(const char \*function, const Eigen::Matrix< T\_y, Eigen::Dynamic, Eigen::Dynamic > &y, const char \*name, T\_result \*result)

Return true if the specified matrix is symmetric.

**Definition:** check\_symmetric.hpp:31

stan::math::check\_multiplicable

bool check\_multiplicable(const char \*function, const T1 &y1, const char \*name1, const T2 &y2, const char \*name2, T\_result \*result)

**Definition:** check\_multiplicable.hpp:15

B\_

Eigen::Matrix< TB, RB, CB > B\_

**Definition:** quad\_form.hpp:49

rev.hpp

value\_of.hpp

A\_

Eigen::Matrix< TA, RA, CA > A\_

**Definition:** quad\_form.hpp:48

stan::math::cols

size\_t cols(const Eigen::Matrix< T, R, C > &m)

**Definition:** cols.hpp:12

Eigen.hpp

stan::agrad::quad\_form

boost::enable\_if\_c< boost::is\_same< TA, var >::value||boost::is\_same< TB, var >::value, Eigen::Matrix< var, CB, CB > >::type quad\_form(const Eigen::Matrix< TA, RA, CA > &A, const Eigen::Matrix< TB, RB, CB > &B)

**Definition:** quad\_form.hpp:129

stan::agrad::quad\_form\_sym

boost::enable\_if\_c< boost::is\_same< TA, var >::value||boost::is\_same< TB, var >::value, Eigen::Matrix< var, CB, CB > >::type quad\_form\_sym(const Eigen::Matrix< TA, RA, CA > &A, const Eigen::Matrix< TB, RB, CB > &B)

**Definition:** quad\_form.hpp:162

stan::math::check\_square

bool check\_square(const char \*function, const Eigen::Matrix< T\_y, Eigen::Dynamic, Eigen::Dynamic > &y, const char \*name, T\_result \*result)

Return true if the specified matrix is square.

**Definition:** check\_square.hpp:24

check\_square.hpp


---

[ Stan Home Page ]

*© 2011–2014,
Stan Development Team.*
